# Construction of a Novel Magnetic Targeting Anti-Tumor Drug Delivery System: Cytosine Arabinoside-Loaded Bacterial Magnetosome

**DOI:** 10.3390/ma6093755

**Published:** 2013-09-03

**Authors:** Qiongjia Deng, Yuangang Liu, Shibin Wang, Maobin Xie, Shenjian Wu, Aizheng Chen, Wenguo Wu

**Affiliations:** 1 College of Chemical Engineering, Huaqiao University, Xiamen 361021, China; E-Mails: dengqiongjia@126.com (Q.D.); sbwang@hqu.edu.cn (S.W.); xmb17010@yahoo.com.cn (M.X.); wsj0840@163.com (S.W.); azchen@hqu.edu.cn (A.C.); wuwenguo@hqu.edu.cn (W.W.); 2 Institutes of Pharmaceutical Engineering, Huaqiao University, Xiamen 361021, China

**Keywords:** cytosine arabinoside, magnetosome, genipin, drug release

## Abstract

To ease the side effects triggered by cytosine arabinoside (Ara-C) for acute leukemia treatment, a novel magnetic targeting anti-tumor drug delivery system was constructed through bacterial magnetosomes (BMs) from *Magnetospirillum magneticum* AMB-1 combined with Ara-C by crosslinking of genipin (GP). The results showed that Ara-C could be bonded onto the membrane surface of BMs effectively through chemical crosslinking induced by dual hand reagents GP. The average diameters of BMs and Ara-C-coupled BMs (ABMs) were 42.0 ± 8.6 and 72.7 ± 6.0 nm respectively, and the zeta potentials (−38.1 ± 9.1) revealed that these systems were stable, confirming the stability of the system. The optimal encapsulation efficiency and drug loading were 89.05% ± 2.33% and 47.05% ± 0.64% respectively when crosslinking reaction lasted for 72 h. The system also presented long-term stability and release behaviors without initial burst release (Ara-C could be released 80% within three months). Our results indicate that BMs have great potential in biomedical and clinical fields as a novel anti-tumor drug carrier.

## 1. Introduction

Cytosine arabinoside (Ara-C) is a pyrimidine anti-metabolism of chemotherapeutic agent, which is most commonly used in the treatment of acute myelogenous leukemia [1]. It can induce cell death of rapidly proliferating cells via blocking the synthesis of nucleic acids from inhibition of the DNA polymerase [2]. However, high doses of Ara-C are cytotoxic can bring about serious side effects for patients, mainly including myelosuppression, digestive tract reactions, hepatotoxicity and neurotoxicity [3,4,5]. Additional, the efficacy of Ara-C is limited by its rapid metabolism and elimination clearance from the body. Since the cytotoxicity associates with the concentration and action time [6], the controlled release of a low dosage of Ara-C at the damage location will provide an efficient way to reduce its severe adverse effects and subsequently enhance the curative effect.

Several approaches have been developed to decrease the side effects of Ara-C administered in chemotherapy over the past decade. For example, agarose hydrogel using layer-by-layer assembly was designed for controlled release of Ara-C. The results showed that the dose released from agarose was sufficient to inhibit fibroblast growth without side effects to the neuronal cells [7]. Moreover, Ara-C-loaded co-matrix microspheres were prepared for long-term, continuous drug administration [8]. Poly(ethylene glycol) (PEG) was used to covalently link with Ara-C, and PEG-Ara-C conjugates showed a increased stability and reduced cytotoxicity as expected [9]. However, the drug loading was relatively low and targeted therapy was not reflected, which would limit the further application of Ara-C.

To solve these problems, bacterial magnetosomes (BMs), superparamagnetic lipid biomembrane-bound nano crystals (Fe_3_O_4_ or Fe_3_S_4_) synthesized by magnetotactic bacteria [10], are used in Ara-C controlled release. The membrane of magnetosomes is rich in phosphatidyl ethanolamine, which makes its surface electronegativity [11]. Moreover, a large amount of protein and amino acids embedded in magnetosome membrane can be used to link bioactive molecules through the crosslinking of dual hand reagents [12]. At present, some researchers have shown interest in BMs as vehicles for antitumor drugs. The BMs have been successfully used as vehicles for antitumor drug doxorubicin (DOX), the DOX-coupled BMs showed cytotoxicity to the cancer cells consistent with that of DOX [13]. In addition, through modifying the membrane surface of BMs with poly-l-glutamic acid, the drug loading was further increased [14]. Compared with artificial magnetic nanoparticles [15,16,17,18,19], BMs are natural magnetic nano-carriers with lipid biomembrane, which can be directly used as a drug carrier. A high drug loading could be achieved by the chemical bond between the drug and the surface of the BMs, while the side effects of Ara-C could be mitigated.

Based on the considerations above, this research aims at obtaining a magnetic targeting anti-tumor drug delivery system constructed by connecting Ara-C on the surface of BMs membrane via chemical crosslinking reaction between amino groups. In our previous study, the MTX nanocomposites with high performances were successfully prepared by using this bacterial magnetosomes [20]. Herein, we developed an Ara-C-loaded BMs (ABMs) delivery system with high drug loading and long-term release behavior by the utilization of a natural dual functional crosslinker genipin (GP) [21]. The characteristics of ABMs, such as morphological properties, zeta potential, FTIR spectra, drug loading, encapsulation efficiency and *in vitro* release were investigated.

## 2. Results and Discussion

### 2.1. The Characterization of ABMs

**Figure 1:**
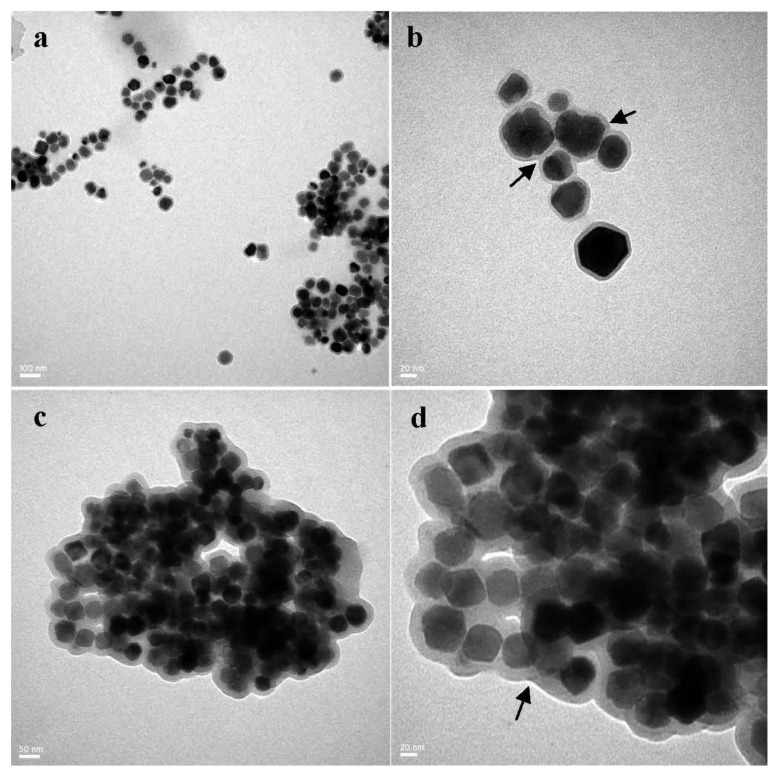
TEM images of bacterial magnetosomes (BMs) and Ara-C-coupled BMs (ABMs): (**a** and **b**: BMs) and (**c** and **d**: ABMs); (**b**) BMs showed the narrow size distribution and uniform lipid membrane; (**d**) Black arrow pointed to thickened and blurred materials surrounding ABMs.

Figure 1 shows the TEM images of BMs and ABMs. Figure 1a revealed that BMs isolated from *M. magneticum* AMB-1 possessed uniform dispersion and narrow size distribution. Figure 1b showed the integrated lipid membrane outside of BMs clearly. As shown in Figure 1c,d, the collected ABMs were surrounded by thickened and blurred materials after the crosslinking reaction. Compared to BMs (42.0 ± 8.6 nm), the average particle size of ABMs increased to 72.7 ± 6.0 nm, which suggested that Ara-C might bind to the membranes of the purified BMs.

As shown in Table 1, the zeta potentials of BMs and ABMs were −30.7 ± 5.9 and −38.1 ± 9.1 mV respectively. The surface electronegativity was ascribed to the abundant phosphatidyl ethanolamine on the BMs membrane. Ara-C is coupled with amino groups in BMs membrane through crosslinking reaction induced by GP, which causes the decrease of positively charged groups and leads to the decrease of the zeta potential in ABMs membrane. These data also proved that BMs and ABMs both have relatively excellent stability.

Based on the results described above, we assume that Ara-C bound to the surface of the membranes of the BMs through chemical crosslinking induced by dual hand reagents GP.

**Table 1:** Size and zeta potential of BMs and ABMs.

Sample	Size (nm)	Zeta potential (mV)
BMs	42.0 ± 8.6	−30.7 ± 5.9
ABMs	72.7 ± 6.0	−38.1 ± 9.1

### 2.2. Examination of the ABMs by FTIR Spectrometer

**Figure 2:**
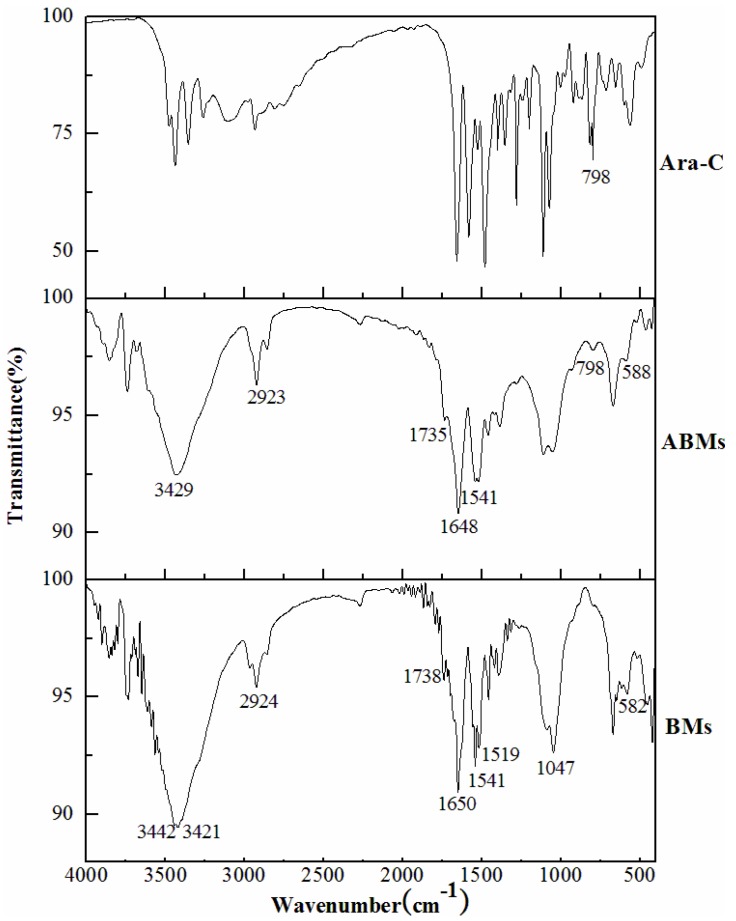
The FTIR spectra of Ara-C, ABMs and BMs.

The Fourier-Transform Infra-Red spectra of Ara-C, ABMs and BMs are shown in Figure 2. The peaks in the spectrum for ABMs were almost identical to those in the spectrum for BMs. The main characteristic absorbance peaks of BMs were located at 3442, 3421, 2924, 1738, 1650, 1541, 1519, 1047 and 582 cm^−1^ respectively. The stretching vibration of amide showed a medium peak at 3442 and 3421 cm^−1^. The methylene stretching vibration was observed at 2924 cm^−1^. The absorbance peak at 1738 and 1650 cm^−1^ respectively were due to carbonyl and alkenyl stretching vibration. The phosphate stretching vibration was found at 1047 cm^−1^ in the wave number range below 1400 cm^−1^. The weak absorbance peak around 582 cm^−1^ was assigned to the typical Fe–O stretching vibration [22].

It is noteworthy that the FTIR of ABMs showed weaker absorbance at both 3429 and 1541 cm^−1^ than that of BMs. The reason is likely due to the reduction of primary amino groups on ABMs. Additionally, the stronger absorbance peak of ABMs at 1735 cm^−1^ than that of BMs may be due to the introduction of carbonyl with the loading of Ara-C. The absorption peaks at 798 cm^−1^ were found in the spectra of both ABMs and Ara-C while the BMs showed no absorbance peak. All these data suggested that Ara-C successfully bound to the lipid membrane surface of the BMs through crosslinking reaction medicated with GP between primary amino groups.

### 2.3. Drug Loading and Encapsulation Efficiency of ABMs

**Figure 3:**
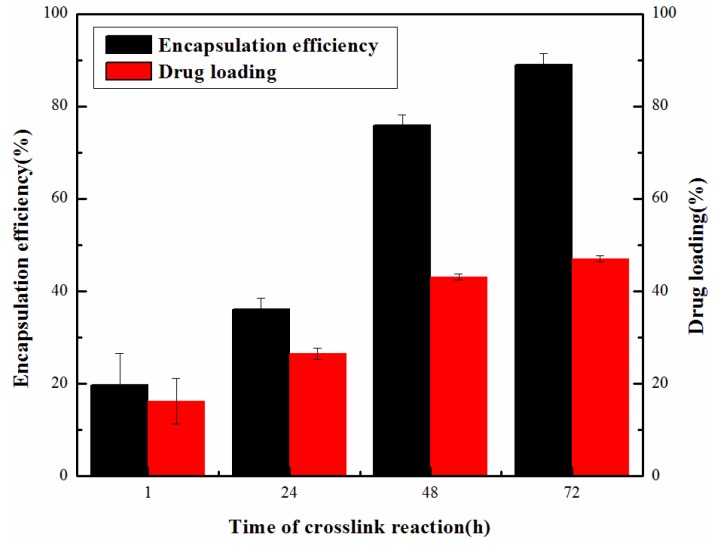
Drug loading and encapsulation efficiency of ABMs prepared with different reaction time by genipin (GP).

It can be seen from Figure 3 that the time of crosslinking reaction significantly influenced the drug loading and encapsulation efficiency of ABMs. When the crosslinking reaction lasted for 72 h, both the encapsulation efficiency and drug loading obtained a maximum value of 89.05% ± 2.33% and 47.05% ± 0.64% respectively. Ara-C was coupled with BMs through chemical crosslinking reaction and physical adsorption. The crosslinking degree of drug mainly depended on the completion level of the crosslinking reaction. Therefore, the crosslinking reaction time was one of the key factors. The longer the crosslinking reaction time, the more Ara-C bound to the BMs due to the more thorough crosslinking reaction.

### 2.4. *In Vitro* Ara-C Release from ABMs

**Figure 4:**
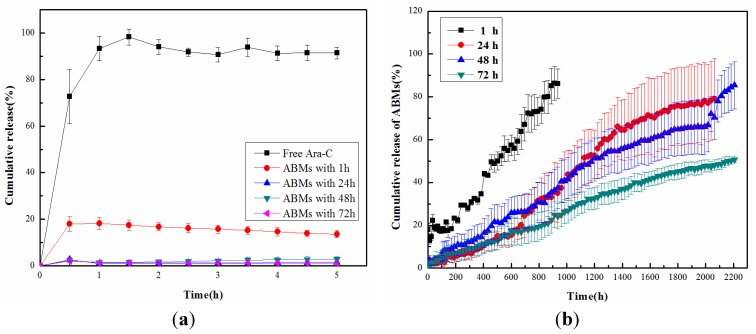
Cumulative release of (**a**) free Ara-C and (**b**) ABMs.

Figure 4 showed the release of Ara-C from ABMs prepared for different crosslinking time in PBS (pH 7.4). As shown Figure 4, free Ara-C almost released completely within less than 5 h, while 80% Ara-C released from ABMs in the next three months. Furthermore, drug burst release behavior was not found in the initial half an hour. When the crosslinking reaction time was shortened to 1 h, more than 80% Ara-C released within 40 days. However, with the extension of crosslinking reaction time, the release rate of Ara-C gradually decreased. Crosslinking for 72 h revealed a minimum release rate that less than 50% Ara-C released within 90 days.

The reason was analyzed as follows: The degradation of BMs membrane is critical for the Ara-C release from ABMs. The longer crosslinking reaction time will lead to the more thorough chemical crosslinking reaction, and then Ara-C will couple with BMs more closely, which accordingly extends the release period. These results confirmed that ABMs showed long-term stability and release behavior, which validated the feasibility of preparing method of Ara-C-loaded BMs introduced in this research.

## 3. Experimental Section

### 3.1. Materials

*Magnetospirillum magneticum* AMB-1 was kindly provided by Professor Pan (Institute of Geology and Geophysics, Chinese Academy of Sciences, Beijing, China). Cytosine arabinoside was purchased from Sunray Pharmaceutical Co., Ltd. (Suzhou, China). Genipin was purchased from Zhixin Biotechnology Company (Fuzhou, China). All other chemicals were obtained from Sinopharm Chemical Reagent Co., Ltd. (Shanghai, China).

### 3.2. Isolation and Purification of BMs

*M. magneticum* AMB-1 were incubated for 48 h in constant temperature incubator at 26 °C. Bacterial cells collected by centrifugation (10,000 rpm, 4 °C, 10 min) were resuspended in PBS (pH 7.4), and disrupted by ultrasonication (300 W, 15 min) using an ultrasonic cell crusher (JY92-2D, Ningbo Scientz Biotechnology Co., Ltd., China). BMs were collected by absorption with a magnet bounded on the bottom of tubes, and cell debris was removed. The BMs sediments were washed by adding PBS (pH 7.4). Following being resuspended in PBS (pH 7.4) and ultrasonic cleaning (40 W, 5 min), BMs were recollected by a permanent magnet, and this procedure was repeated for 5–10 times. In order to determine whether any protein still attached on the surface of BMs, the supernatant absorption value of purified BMs was measured at 280 and 260 nm using U-vis spectrophotometer (UV-1600PC, Mapada Instruments Co., Ltd., Shanghai, China), and the amount of protein (A) was calculated using the following equation: A (g/L) = 1.45 × OD_280_ − 0.74 × OD_260_. Moreover, purification level of BMs was evaluated using optical microscope.

### 3.3. Preparation of the ABMs

Purified BMs were distributed in PBS (pH 7.4) and dispersed completely with ultrasonication (50 W, 5 min). Following an addition of 1 mg/mL Ara-C solution, BMs suspension was treated with ultrasonic bathing for 5 min. Then 1% GP sulotion was added into above BMs mixture, which was treated with ultrasonic bathing 10 times (working 1 min with interval of 5 min every time) and then placed in an incubator shaker (KYC 100B, Shanghai Fuma Test Equipment Co., Ltd., Shanghai, China) at 60 rpm, 37 °C for 1, 24, 48, 72 h to achieve an adequate reaction.

### 3.4. Observation of the ABMs by Transmission Electron Microscopy

Purified BMs and ABMs supernatant prepared by dispersing in PSB were dropped on the cropper grids. After natural drying, the grids were examined and recorded by transmission electron microscope (H-7650, Hitachi, Ltd., Tokyo, Japan).

### 3.5. Size Distribution and Zeta Potential of the ABMs and BMs

The particle size of ABMs and BMs was obtained by counting 500 ABMs and BMs in TEM images to calculate size distribution. The zeta potential was determined by Zetasizer (ZEN3600, Malvern Instruments Ltd., Worcestershire, UK).

### 3.6. Examination of the ABMs by FTIR Spectrometer

After freeze drying with a vacuum freeze dryer (FD-1B-50, Boyikang Lab Instrument Co., Ltd., Beijing, China), Ara-C, ABMs and BMs mixed with KBr were respectively grinded into fine powder. FTIR spectrum of the above samples was recorded on a FTIR spectrometer (NICOLET iS10, Thermo Fisher Scientific, Waltham, MA, USA) in the wave number range of 4000–400 cm^−1^.

### 3.7. Drug Loading and Encapsulation Efficiency of ABMs

After washing with magnet absorption in conjunction with vortex mixing until the supernatant solution showed no color to eliminate superfluous Ara-C and GP, the ABMs were completely distributed in 5 mL PBS. 0.2 mL ABMs suspensions was dissolved in breaking-membrane liquid consisted of 1.2 mL 36%–38% hydrochloric acid and 3.6 mL 70% ethanol solution stand for 1 h, and the absorption value was measured at 286 nm. The concentration of Ara-C completely released from ABMs was calculated through the following equation of the standard curve: *y* = 18.0798*x* − 0.2156, where *y* value was concentration of Ara-C (μg/mL) and *x* value was absorption of Ara-C in breaking-membrane liquid at 286 nm. Thus the amount of Ara-C coupled to ABMs was obtained. All assays were performed in triplicate.

According to China Pharmacopoeia (2010 version), drug loading and encapsulation efficiency were respectively calculated using the following equations: drug loading (%) = (*W*_1_/*W*_2_) × 100%, encapsulation efficiency (%) = (*W*_1_/*W*_3_) × 100%, where *W*_1_ is the amount of Ara-C coupled to ABMs, *W*_2_ is the amount of ABMs, *W*_3_ is the amount of Ara-C coupled to ABMs and in reaction solution.

### 3.8. *In Vitro* Ara-C Release from ABMs

The ABMs dispersed in PBS was placed in an incubator shaker at 60 rpm, 37 °C with magnet absorption and the absorption value of Ara-C in the supernate was measured at 271.6 nm every 12 h using U-vis spectrophotometer (UV-1600PC, Mapada Instruments Co., Ltd., Shanghai, China). The concentration of Ara-C was calculated through the following equation of the standard curve: *y* = 26.4272*x* − 0.1996, where y value was concentration of Ara-C (μg/mL) and *x* value was absorption of Ara-C in PBS at 271.6 nm. *In vitro* Ara-C release curve was obtained. All assays were performed in triplicate.

## 4. Conclusions

Our results clearly show that BMs isolated from *M. magneticum* AMB-1 can be used as a carrier of anticancer drugs with the help of proteins and other functional groups embedded in its membrane. Through crosslinking stimulated by the natural biological agent GP, Ara-C was bound to the membranes of BMs which showed a strongly enhanced controlled drug release effect thereby relieving the severe side effects of the drug. Our preliminary studies demonstrate the great potential for future biomedical applications of BMs. 
